# Computerized general practice based networks yield comparable performance with sentinel data in monitoring epidemiological time-course of influenza-like illness and acute respiratory illness

**DOI:** 10.1186/1471-2296-11-24

**Published:** 2010-03-22

**Authors:** Carla Truyers, Emmanuel Lesaffre, Stefaan Bartholomeeusen, Bert Aertgeerts, René Snacken, Bernard Brochier, Fernande Yane, Frank Buntinx

**Affiliations:** 1 Department of General Practice, Katholieke Universiteit Leuven, Leuven, Belgium; 2 L-Biostat, Katholieke Universiteit Leuven, Leuven, Belgium; 3 Department of Biostatistics, Erasmus University Rotterdam, Rotterdam, the Netherlands; 4 National Influenza Centre, Scientific Institute of Public Health, Brussels, Belgium; 5 Research Institute Caphri, Maastricht University, Maastricht, the Netherlands

## Abstract

**Background:**

Computerized morbidity registration networks might serve as early warning systems in a time where natural epidemics such as the H_1_N_1 _flu can easily spread from one region to another.

**Methods:**

In this contribution we examine whether general practice based broad-spectrum computerized morbidity registration networks have the potential to act as a valid surveillance instrument of frequently occurring diseases. We compare general practice based computerized data assessing the frequency of influenza-like illness (ILI) and acute respiratory infections (ARI) with data from a well established case-specific sentinel network, the European Influenza Surveillance Scheme (EISS). The overall frequency and trends of weekly ILI and ARI data are compared using both networks.

**Results:**

Detection of influenza-like illness and acute respiratory illness occurs equally fast in EISS and the computerized network. The overall frequency data for ARI are the same for both networks, the overall trends are similar, but the increases and decreases in frequency do not occur in exactly the same weeks. For ILI, the overall rate was slightly higher for the computerized network population, especially before the increase of ILI, the overall trend was almost identical and the increases and decreases occur in the same weeks for both networks.

**Conclusions:**

Computerized morbidity registration networks are a valid tool for monitoring frequent occurring respiratory diseases and the detection of sudden outbreaks.

## Background

In a time where natural epidemics such as the H_1_N_1 _flu can spread easily from one region to another, where concerns about industrial or other pollutants and potential bio terrorist agents are vast, well functioning surveillance schemes for diseases might become indispensable allies [1,2]. One of the most important settings in the surveillance of diseases is general practice (GP). GPs are probably one of the first parties to be confronted with an unexpected rise in disease frequency[3,4]. With regard to bioterrorist attacks they are considered as frontline soldiers in this new form of warfare [5].

Early detection could nowadays be supported by the use of the electronical medical records (EMR), which can be programmed to immediately give an alert when there is an unexpected rise in disease[6]. Indeed, the shift from written to computerized medical records enables an easier access to individual health data. Health information is only a press of a button away. This makes computerized sentinel networks an important source of information.

A question that remains to be answered is that of quality and validity. Are data from GP-based computerized morbidity systems good enough to monitor diseases and thereby possibly function as an early warning system?

Influenza-like illness (ILI) and acute respiratory infections (ARI) are chosen as diseases of interest for several reasons. Diagnoses are based on clinical signs and symptoms and they are very well known by GPs. General Practice is also the only valid setting for epidemiological information on ILI and ARI [7]. Both diseases have a considerable morbidity rate [8] with significant impact on daily activities and they are very well documented in literature.

In Belgium, there are several systems which can provide primary care data. However, only one computerized general practice based registration network is fully established. Intego was founded in 1994 and provides, among other data, data on incidences of all diseases in primary care. The European Influenza Surveillance Scheme (EISS) is a case-specific surveillance network in which 28 member countries collect and exchange timely information on influenza activity in Europe and provides early warning in case of an outbreak[9]. The Belgian branch of the network, also collects information on ARI and was a pioneer of EISS [10,11]. In 2008 EISS was changed to EISN (European Influenza Surveillance Network).

This article describes the comparison of data generated by the Intego database and compares it to EISS data as a possibility to validate continuous sentinel networks as possible surveillance tools.

## Study population

The Intego network is the first computerized network of voluntary sentinel general practitioners in Flanders, the northern, Dutch speaking part of Belgium [12,13]. The network is organised within the department of General Practice of the Katholieke Universiteit Leuven and provides data on incidences and prevalence of all diseases in Flanders, but also on laboratory tests and drug prescriptions from 1994 onward. Data can be classified by ICPC-2 (International Classification of Primary Care), which also incorporates codes for patient reasons for encounter, symptoms and ill-defined conditions[14]. However, also more detailed diagnostic categories are available. GPs are included in the network on the basis of different quality criteria to maximize the validity and reliability of the data. In 2007 the Intego database includes 86 GPs with over 2.1 million diagnoses, and covers almost 2% (practice population of 120.000) of the population in Flanders, the northern part of Belgium. The population is representative for the Flemish population with regard to age, sex and socio-economical factors [15]. The project was approved by the Ethical review board of the Faculty of Medicine of KULeuven. Similar networks can be found in Europe [16].

The clinical surveillance of influenza by the EISS in Belgium is based on written registration forms by voluntary sentinel general practitioners[17]. It is organised within the Scientific Institute of Public Health. The network was started in 1985 and includes 60 GPs who are specifically monitoring influenza and acute respiratory infections (about 50% in Flanders, 45% Walloon, and 5% in Brussels). Since the end of 2007 the network was increased to 200 GPs. During the influenza season (week 40 to week 20 of the following year), a first GP network collects data on influenza activity during the previous week. Additionally, nose/throat swabs are performed to identify the influenza virus by a second sentinel network of GPs and also by laboratories essentially from universities. The identification of flu strains is performed by the National Centre of Influenza from the Scientific Institute of Public Health unit of Virology.

A comparison of the geographical spread of both networks revealed that in 2003 both networks were spread over 40 municipalities, for Intego all situated in Flanders and for the EISS about 50% located in Flanders. In nine of these, both networks were represented.

## Methods

The EISS defines ILI as all acute respiratory infections accompanied by flu-like symptoms, i.e. sudden onset, fever, myalgias and respiratory symptoms. ARI is defined as any infection involving the respiratory tract, with or without fever, which lasts one to two weeks. The Intego database uses ICPC-2 code R-80 (influenza defined as myalgia and cough without abnormal respiratory physical signs other than inflammation of nasal mucous membrane and throat, plus three or more of the following: sudden onset (within 12 hours); rigors/chills/fever; prostration and weakness; influenza in close contacts; influenza epidemic; or viral culture/serological evidence of influenza virus infection) as a measure of ILI, and H71 (otitis media), R74 (acute upper respiratory infection including rhinitis, rhinopharyngitis, pharyngitis), R75 (sinusitis), R76 (acute tonsillitis) R77 (acute laryngitis, tracheitis), R78 (acute bronchitis, bronchiolitis) and R81 (pneumonia) as measures of ARI (table 1).

**Table 1:** Measurement of acute respiratory infections and accompanying ICPC-2 coding in the Intego network

ARI diagnoses	ICPC-2 code
rhinitis	R74
rhinopharyngitis	R74
pharyngitis	R74
amygdalitis	R76
sinusitis	R75
middle ear infection	H71
laryngitis	R77
tracheitis	R77
bronchitis	R78
bronchiolitis	R78
pneumonia	R81
bronchopneumonia	R81

Although the Intego registry usually works on the basis of an estimated practice population, the denominator populations are based on the number of consultations for reasons of comparability. Therefore data for the analyses is percentage of consultations for ILI and ARI compared to all consultation reasons. Only in the figures, data on absolute numbers are also shown. We have split up the data according to the influenza season (week 40 to week 20 of the following year). Since Intego only uses ICPC-2 coding from 1999 onward, only data for the seasons 1999-2000 to 2002-2003 were included in our study.

Two questions are of interest. First, the overall difference in frequency between the two networks. This difference can be expected since Intego GPs are not focussing on registering specific diagnoses. We will take the difference in values (Intego minus EISS) for each influenza season, thereby also reducing the serial dependence[18]. The Student's t-test on the difference between the two time series will be used to evaluate whether they differ overall in the mean, justified partly because of the central limit theorem. To account for the possible effect of dependency in the data, we repeat the analysis after correction for dependent observations by correcting the degrees of freedom used in the t-test with the number of lags still exhibiting autocorrelation [19]. Secondly, and maybe more importantly, next to looking at the overall difference, we want to see whether the rising and declining trends in both networks are comparable. A similar overall trend (as referred to in question 1), but with totally different seasonal curves will not indicate good validity to detect rapid changes in case of surveillance objectives. Therefore increasing/decreasing trends in one network should coincide with increases/decreases in the other network at the same time. In case the two trends differ, it will be examined whether there is a better association between the two time series if various time lags are taken by means of cross correlation coefficients. We use the SAS arima procedure to calculate the cross correlations between both networks up to lag 5, each lag representing a difference of one week[20]. A cross correlation on the original time series can be interpreted as how many lags the EISS data have to shift in order to match the Intego data. The Intego time series is used as the input data set, therefore a correlation at lag minus 1 represents the relation between the Intego data at week_n _and the EISS data at week_n+1_. A cross correlation on the time series which have been differenced indicates the relation between the changes in both series. The Intego time series is again used as the input data set, therefore a correlation at lag minus 1 represents the relation between the changes in the Intego data at week_n _and the changes in the EISS data at week_n+1_. In case there is a positive cross correlation present at a positive lag, the changes in the EISS data occur more rapid than for the Intego data. Significant negative correlations would mean an opposite relationship between both networks.

## Results

**Figure 1:**
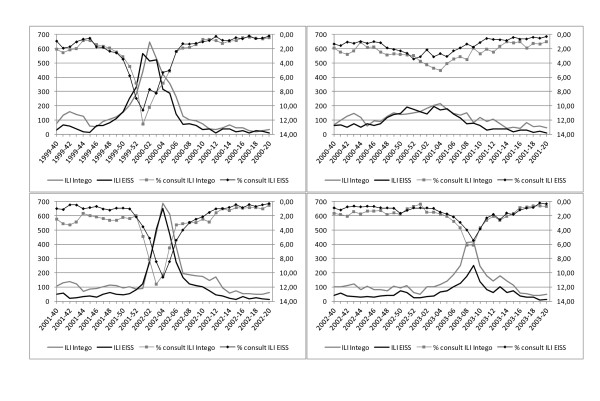
**ILI data from EISS (black) and Intego data (grey) from influenza season 1999-2000 to 2002-2003**. The percentage of ILI compared to all consultations is presented with a marker and refers to the right axis, which is presented in reverse order. The absolute number of ILI is presented without marker and refers to the left axis.

The data for influenza like illness for the seasons 1999-2000, 2000-2001, 2001-2002 and 2002-2003 are presented in figure 1. The vertical axis plots the percentage of consultations and absolute numbers for ILI on a weekly basis (horizontal axis).

Visual inspection shows that the curves for both networks are in high agreement. The rise in frequency for ILI occurs around the same time. In most seasons ILI starts with a period of only a small number of cases, followed by a sharp increase in frequency to reach its peak after about one month and then goes down again to a small number of cases. Season 2001-2002 is somewhat unusual as no typical influenza peak was discovered compared to the other seasons.

**Figure 2:**
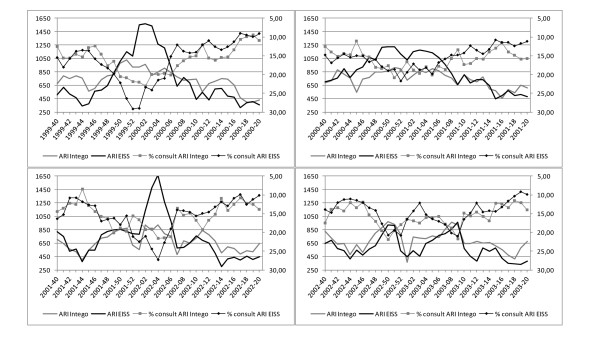
**ARI data from EISS (black) and Intego data (grey) from influenza season 1999-2000 to 2002-2003**. The percentage of ARI compared to all consultations is presented with a marker and refers to the right axis, which is presented in reverse order. The absolute number of ARI is presented without marker and refers to the left axis.

The percentages of consultations and absolute numbers for ARI are shown in figure 2.

The curves are visually very similar for both networks. ARI data have a less smooth profile and do not show a marked rise, peak and decrease pattern which is typical for the ILI data. They are higher in frequency compared to ILI, at all time points.

In general the overall ARI data are not significantly different for both networks. For the ILI data however there appears to be a significant difference between both networks in most seasons. The means of the resulting distributions for ILI are all positive, indicating an overall higher rate for the Intego data in comparison to the EISS data. This could be attributable to the higher frequency in Intego in the period before the real start of the influenza rise.

When accounting for dependency in the data, the conclusions remained the same.

The results regarding the rising/declining trends are shown in table 2. The highest cross correlations of both the original and differenced series are shown.

**Table 2:** Cross correlation coefficients between the Intego and EISS network at different lags for acute respiratory infections and influenza like illness

	Lag (cross-correlation)*	
	ILI	ARI
**Original series**		
1999-2000	**0 **(0.96)/**1 **(0.94)	**0 **(0.84)/**1 **(0.86)
2000-2001	**0 **(0.80)/**1 **(0.82)	**0 **(0.65)/**-1 **(0.63)
2001-2002	**0 **(0.89)/**-1 **(0.92)	**0 **(0.77)/**1 **(0.77)
2002-2003	**0 **(0.92/**-1 **(0.88)	**0 **(0.82)/**-1 **(0.79)
**Differenced series (lag 1)**		
1999-2000	**0 **(0.71)/**1 **(0.61)	**1 **(0.41)
2000-2001	**3 **(0.37)	**7 **(0.35)
2001-2002	**0 **(0.71)/**-1 **(0.81)	**-7 **(0.49)
2002-2003	**0 **(0.73)	**0 **(0.44)

* (, for s = -L to L, where L is the number of lags.).

The original data for ILI and ARI show high to very high cross correlations at lag 0, indicating very significant linear relationships between both series. This finding is in accordance with the visual inspection of the figures as stated above. High cross correlations are also found at lag minus 1 and 1, providing an indication of significant autocorrelation at lag 1, which is not uncommon in case of infectious diseases. The differenced data reveal high correlations at lag 0 for the ILI data (except for season 2000-2001), indicating increases and decreases in the Intego data coincide respectively with increases and decreases in the EISS data. For the ARI data we found high cross correlations mostly at lag 0, also indicating significant linear relationships between both series. The cross correlations on the differenced series were low. Since these ARI data are more variable than the ILI data (more ups and downs) high correlations are not expected as compared to ILI.

## Discussion

Intego can be regarded as a proxy of EISS in examining relative frequency of ILI and ARI as a function of time. Visual comparison of data from the Intego and EISS network revealed almost identical curves for ILI and very similar ones for ARI. From the statistical analysis it is clear that the overall frequency data for ARI are the same for both networks, the overall trends are similar, but that the increases and decreases in frequency do not occur in exactly the same weeks. For ILI, the overall rate was slightly higher for the Intego population, especially before the increase of ILI, the overall trend was almost identical and the increases and decreases in frequency occur in the same weeks for both networks. We expected, however, to find lower rates derived from the Intego database because of the absence of both a case definition and an emphasis on ILI and ARI registration [21]. Therefore the overall higher frequency for ILI in the Intego database was surprising. There could be several reasons for this. Intego registrators have to pass a number of strict quality assessments before they can join the group of active registrators. They have to register a sufficient number of diagnoses per patient before they can enter into the registry.

Comparisons of this sort might be one of the possibilities to assess the quality of registration systems as possible surveillance tools. The presented method can be seen as a validity measurement predefining a well-established network as a gold standard [22]. From classical test theory it is proven that the reliability of an instrument is at least as high as its validity, in other words reliability is a necessary prerequisite for validity, both prerequisites when discussing the quality of instruments.

Routinely collected computerized morbidity data have several important advantages for surveillance purposes. Syndromic surveillance allows the earliest possible identification of increased disease frequency [23]. In case of the Intego network, syndromic surveillance can be extended to all diseases which present themselves in primary care, which might provide an advantage in case of new biological threats, new pollutants etc. Not only respiratory illnesses but, also for example neurological, gastroenterological or dermatological conditions that might be of interest. An additional advantage of routine broad-spectrum registries is that relations of an outbreak with specific subgroup of age and sex, but also with co-morbid diseases, can easily be studied and that consequences of the disease of interest can be identified by the use of a (prospective or retrospective) cohort design.

## Conclusion

Data from GP-based broad-spectrum computerized morbidity systems provide a valid and reliable way to monitor infectious diseases. The main advantage of case specific sentinel networks is the extensiveness by which they can investigate specific topics related to the diseases: identification of strains, monitoring GP's workload etc. The main advantage of computerized sentinel networks is the ease by which a large quantity of information can be processed and analyzed in a short amount of time. One can easily focus on a different disease or topic (for example evolution of depression or drug prescriptions).

## Competing interests

The authors declare that they have no competing interests.

## Authors' contributions

CT designed the study and wrote the first draft of the manuscript. SB, RS, BB and FY performed the data collection. CT and EL undertook the statistical analysis. BA and FB participated in the design of the study. All authors contributed to and have approved the final manuscript.

## Pre-publication history

The pre-publication history for this paper can be accessed here:

http://www.biomedcentral.com/1471-2296/11/24/prepub

## References

[B1] Jarup L Hazards of heavy metal contamination Br Med Bull 2003 68 167 182 10.1093/bmb/ldg032 14757716

[B2] Wise R Bioterrorism: thinking the unthinkable The Lancet 1998 351 1378 10.1016/S0140-6736(05)79441-1 9593406

[B3] Henderson DA The Looming Threat of Bioterrorism Science 1999 283 1279 1282 10.1126/science.283.5406.1279 10037590

[B4] Gerberding JL Hughes JM Koplan JP Bioterrorism Preparedness and Response: Clinicians and Public Health Agencies as Essential Partners JAMA 2002 287 898 900 10.1001/jama.287.7.898 11851584

[B5] Varkey P Poland GA Cockerill FR Smith TF Hagen PT Confronting Bioterrorism: Physicians on the Front Line Mayo Clinic Proceedings 2002 77 661 672 10.4065/77.7.661 12108604

[B6] Jormanainen V Jousimaa J Kunnamo I Ruutu P Physicians' database searches as a tool for early detection of epidemics Emerg Infect Dis 2001 7 474 476 11384534 10.3201/eid0703.010324 PMC2631811

[B7] Sauro A Barone F Blasio G Russo L Santillo L Do influenza and acute respiratory infective diseases weigh heavily on general practitioners daily practice? The European Journal of General Practice 2006 12 34 36 10.1080/13814780600757153 16945870

[B8] Sessa A Costa B Bamfi F Bettoncelli G D'Ambrosio G The incidence, natural history and associated outcomes of influenza-like illness and clinical influenza in Italy Fam Pract 2001 18 629 634 10.1093/fampra/18.6.629 11739352

[B9] Influenza Surveillance: Why? Vaccine 2006 24 6770 6775 10.1016/j.vaccine.2006.06.073 17167881

[B10] Snacken R Manuguerra JC Taylor P European Influenza Surveillance Scheme on the Internet Methods of Information in Medicine 1998 37 266 270 9787627

[B11] EISS 2010 http://www.ecdc.europa.eu/en/activities/surveillance/EISN/Pages/home.aspx

[B12] Bartholomeeusen S Kim CY Mertens R Faes C Buntinx F The denominator in general practice, a new approach from the Intego database Fam Pract 2005 22 442 447 10.1093/fampra/cmi054 15964863

[B13] Bartholomeeusen S Truyers C Buntinx F Diseases in general practice in Flanders 2004 Leuven: Academisch Centrum voor Huisartsgeneeskunde, K.U.Leuven

[B14] Okkes IM Becker HW Bernstein RM Lamberts H The March 2002 update of the electronic version of ICPC-2. A step forward to the use of ICD-10 as a nomenclature and a terminology for ICPC-2 Fam Pract 2002 19 543 546 10.1093/fampra/19.5.543 12356710

[B15] Bartholomeeusen S Morbidity research in primary care, using semi-automatic data collection from electronic medical records in general practices in Flanders 2008 Leuven: Acco

[B16] Deckers JG Paget WJ Schellevis FG Fleming DM European primary care surveillance networks: their structure and operation Fam Pract 2006 23 151 158 10.1093/fampra/cmi118 16464870

[B17] Uphoff H Cohen JM Fleming D Noone A Harmonisation of national influenza surveillance morbidity data from EISS: a simple index Eurosurveillance 2003 8 156 164 12941981 10.2807/esm.08.07.00420-en

[B18] Zeger SL Irizarry R Peng RD On time series analysis of public health and biomedical data Annual Review of Public Health 2006 27 57 79 10.1146/annurev.publhealth.26.021304.144517 16533109

[B19] Priestley MB Spectral Analysis and Time Series 1981 New York: Academic Press

[B20] SAS® 9.1.3 2007 Cary, NC, USA, SAS Institute inc.

[B21] Thursky K Cordova SP Smith D Kelly H Working towards a simple case definition for influenza surveillance J Clin Virol 2003 27 170 179 10.1016/S1386-6532(02)00172-5 12829039

[B22] Karras DJ Statistical methodology: II. Reliability and validity assessment in study design, Part B Acad Emerg Med 1997 4 144 147 10.1111/j.1553-2712.1997.tb03723.x 9043544

[B23] Lazarus R Kleinman K Dashevsky I DeMaria A Platt R Using automated medical records for rapid identification of illness syndromes (syndromic surveillance): the example of lower respiratory infection BMC Public Health 2001 1 9 10.1186/1471-2458-1-9 11722798 PMC60002

